# Cubic Cesium Lead Bromide Stabilized by Ethylammonium Incorporation

**DOI:** 10.1021/acs.inorgchem.5c02183

**Published:** 2025-08-05

**Authors:** Maksim Tabatadze, Aleksandra D. Valueva, Hope A. Long, Sergei A. Novikov, Yiping Zhao, Vladislav V. Klepov

**Affiliations:** a Department of Chemistry, 1355 University of Georgia, Athens, Georgia 30602, United States; b Department of Physics and Astronomy, 1355 University of Georgia, Athens, Georgia 30602, United States

## Abstract

Lead halide perovskites are widely studied as semiconductors for optoelectronic applications, in particular, for solar cells and room temperature radiation detectors. Although many lead halide perovskite compounds exhibit excellent properties, achieving their optimal performance is hindered by structural distortion from an ideal cubic to orthorhombic or monoclinic symmetries. To address this issue and stabilize the cubic structure in CsPbBr_3_, we employed ethylammonium cation to form a series of Cs_*x*_(EtNH_3_)_1–*x*_PbBr_3_ solid solutions. We found that, unlike the widely utilized formamidinium CH­(NH_2_)_2_^+^ that has been previously reported to stabilize up to ≈30% Cs^+^ content, larger ethylammonium EtNH_3_^+^ cation increases the incorporation of Cs^+^ up to 65% while maintaining the cubic structure. The resulting samples exhibit increased stability in humid air and show only slight signs of decomposition onset in 24 h. Optical measurements determined a bandgap of 2.27 eV for Cs_0.65_(EtNH_3_)_0.35_PbBr_3_, which is almost the same as that of pristine CsPbBr_3_, indicating that the optoelectronic properties of the resulting phase remain largely unchanged. Electrical property measurements confirm a large resistivity of 40.1 MΩ·cm in the resulting phase for a pressed pellet sample.

## Graphical abstract

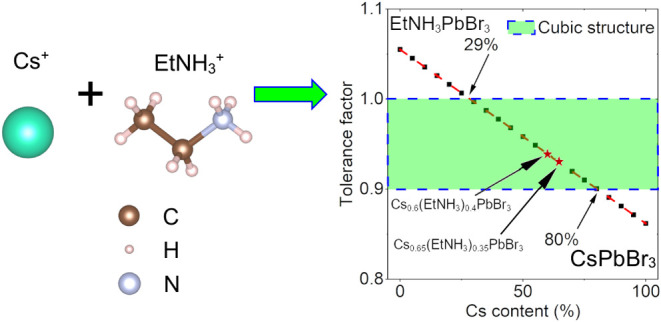

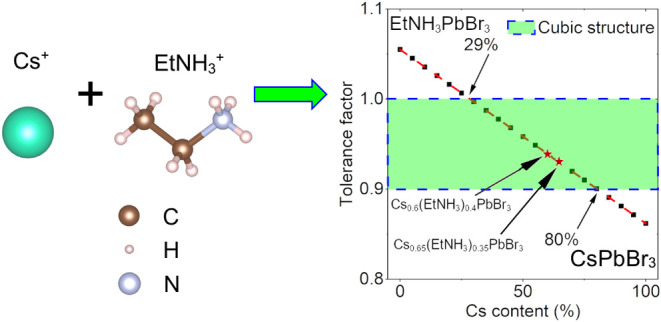

## Introduction

Lead halide perovskites have garnered significant attention in the fields of optoelectronics and photovoltaics^1–3^ due to their exceptional properties,^4^ including tunable bandgaps,^5,6^ high absorption coefficients, and long charge carrier lifetimes,^7,8^ which make them suitable for next-generation optoelectronic devices.^9^ They have shown promise in a wide range of applications, including solar cells,^10–18^ photodetectors,^19–25^ light-emitting diodes,^26–28^ lasers,^29–31^ and radiation detectors.^32–34^ Their unique properties stem from their structure, generally described as a cubic 3D framework with the *ABX*_3_ composition.^35^ In this formula, *A* is a monovalent cation, such as Cs^+^, methylammonium (CH_3_NH_3_^+^), or formamidinium (NH_2_CHNH_2_^+^), *B* is typically a divalent metal cation, Pb^2+^ or Sn^2+^, and *X* stands for a halide anion (Cl^–^, Br^–^, or I^–^).^36^ The perovskite structure consists of corner-sharing *BX*_6_ octahedra, with the *A* cation located in the space between these octahedra, stabilizing the 3D lattice. The choice of cation and halide allows tuning of the perovskite’s properties, such as bandgap,^5^ stability,^15,37–39^ and charge transport characteristics.^7,8^

Although halide perovskites offer significant advantages, many compounds in this system face challenges in achieving a stable cubic structure,^33,40^ which is often critical for optimal device performance.^41^ Structural instability limits their practical application, as phase transitions and degradation can reduce efficiency, long-term performance, and make device fabrication challenging due to the formation of phase boundaries. For example, although CsPbBr_3_ exhibits an outstanding performance as a radiation detector, its synthesis is challenging because of the phase transitions that it undergoes upon cooling after a typical Bridgman or solution growth. To address this issue, mixed-cation lead halide perovskites have been employed to fine-tune structural stability and electronic properties by incorporating various organic and inorganic cations.^15,33,37^ Although some organic cations, such as methylammonium and formamidinium, are commonly employed to stabilize the cubic structures, their relatively small sizes require a significant organic component incorporation. For example, a large formamidinium cation allows for accommodating up to ∼30% of Cs^+^ in (FA)_1–*x*_Cs_*x*_PbBr_3_ before the structure starts to distort from cubic.^42,43^ However, large fractions of organic cations reduce the resistance of the resulting phases to oxygen and moisture.^44^ To minimize this instability and to reduce the amount of the organic cation, one can employ larger cations, which would enable achieving a larger effective radius of the *A*-site when using larger fractions of Cs^+^ cation. Although ethylammonium is among the largest cations that can be incorporated into the perovskite structure,^45^ its solid solutions with Cs^+^ remain underexplored. Particularly, the solid solutions of cesium (Cs^+^) and ethylammonium (EtNH_3_^+^) in lead bromide perovskites Cs_*x*_(EtNH_3_)_1–*x*_PbBr_3_ present a promising solid solution system for the growth of radiation detector crystals due to optimal bandgap and charge carrier properties, based on reported incorporations of dimethylammonium, guanidinium and formamidinium cations into cesium lead halide lattice.^43,46^ However, the stability of compounds in this series has not been studied.

In this report, we synthesized and characterized a series of Cs_*x*_(EtNH_3_)_1–*x*_PbBr_3_ solid solutions with varying Cs^+^ contents. We studied the impact of Cs^+^ and EtNH_3_^+^ incorporation on the structural, optical, and electrical properties of the material, focusing on the cubic phase stability and suitability for optoelectronic applications. The goal is to determine the composition range that maintains a stable cubic phase and investigate its potential for radiation detection applications.

## Experimental Section

### Starting Materials

Ethylamine (Sigma-Aldrich, 66.0–72.0%) and hydrobromic acid (Thermo Scientific, 48%) were used without further purification. Cesium bromide was obtained by reacting cesium carbonate (Thermo Scientific, 99%) with concentrated HBr (Thermo Scientific, 48%) in DI water. Crystalline CsBr was precipitated by adding acetone until no more visible precipitate formed. The precipitate was vacuum filtered and dried in a vacuum oven at 100 °C overnight. Lead bromide (Thermo Scientific, >98%) was washed with deionized water containing a few drops of hydrobromic acid, then filtered and dried in a vacuum oven overnight.

### Synthesis

#### EtNH_3_Br

Five mL of ethylamine solution was mixed with 5 mL of hydrobromic acid. The solution was stirred and then evaporated at 60 °C to obtain colorless crystals. The mixture was cooled to room temperature; then the crystals were collected by vacuum filtration and dried in a desiccator under dynamic vacuum overnight. The phase purity of the crystals was confirmed by PXRD analysis. Yield: 2.378 g (43% based on HBr).

#### Cs_*x*_(EtNH_3_)_1–*x*_PbBr_3_ Solid Solutions

Samples were prepared by grinding stoichiometric amounts of CsBr and EtNH_3_Br with the addition of 10% excess EtNH_3_Br over CsBr and 0.431 g PbBr_2_. The ground powder was pelletized, sealed in a silica tube under a dynamic vacuum, and annealed at 270 °C for 16 h. The samples were reground and reannealed two times for each composition until PXRD confirmed the formation of a single-phase product. Due to their hygroscopic nature, the samples were stored in an Ar-filled glovebox and taken out only for characterization.

#### Powder X-ray Diffraction Measurements

Powder X-ray diffraction (PXRD) data were measured on a Bruker D2 Phaser X-ray diffractometer using a Cu Kα radiation source and a 1D-linear Lynxeye XE-T position-sensitive detector to check phase purity. X-ray source operated at 30 kV/10 mA. Diffraction patterns were recorded from 5 to 65 ° 2θ with 0.1 mm slits and knife edge installed. A typical scan rate was 0.25 s/step with a step size of 0.02 deg. Lattice parameter *a* was calculated from PXRD peak positions using Le Bail fit method in FullProf Suite 5.20 program.^47^

#### Conductivity Measurements

The Cs_*x*_(EtNH_3_)_1–*x*_PbBr_3_ (*x* = 0.6 and 0.65) powders were ground using a mortar and pestle for several minutes to form fine powders. A 0.25 in. diameter cylindrical stainless steel die set (MTI Corporation) was filled with 0.120–0.234 g of sample between two stainless steel dies. The upper die was pressed with a mechanical press in a glovebox to isolate the sample from the environment. Then, a pressure of ≈930 MPa was applied in the air overnight to obtain opaque pellets. After obtaining 0.86–1.86 mm thick pellets, they were stored in the glovebox before further characterization. Gold electrodes were brushed onto the opposite surfaces of the pellet. A copper wire connected the electrodes to the outer circuit, and the device was encapsulated in paraffin wax. The I–V characteristic curves were measured by a Keithley 6517B electrometer under dark conditions, where resistivity was calculated in the bias range of 0 to 100 V.

#### Optical Spectroscopy

Diffuse reflectance spectra were collected at room temperature using a Shimadzu UV-2450 (Kyoto, Japan) spectrometer in a wavelength range from 200 to 800 nm. BaSO_4_ was employed as a nonabsorbing reflectance reference for calibration. Experimental reflection data was transformed into absorption spectra based on Kubelka–Munk theory and optical bandgaps were extracted.^48^ The Kubelka–Munk equation is

$$F\left(R\right)=\frac{K}{S}=\frac{{\left(1-R\right)}^{2}}{2R}$$ 1

where *R* is the reflectance, *F*(*R*) is the Kubelka–Munk function and *K* and *S* are the absorption and scattering coefficients, respectively. The Raman spectra were collected using a Renishaw inVia confocal Raman microscope system with a 633 nm laser. ATR-FTIR (attenuated total reflectance - Fourier transform infrared) spectra were recorded on a Nicolet iS50 spectrometer equipped with DTGS-ATR detector. The scan parameters used were 32 scans at 4.0 cm^–1^ resolution with prior collection of background spectra and analyzed using the OMNIC Software.

#### Scanning Electron Microscopy (SEM)

SEM images were acquired using a Thermo Fisher Teneo FE-SEM operated at 20 kV with a CBS detector.

#### Nuclear Magnetic Resonance (NMR) Spectroscopy

NMR spectra were recorded for Cs_0.6_(EtNH_3_)_0.4_PbBr_3_ and Cs_0.65_(EtNH_3_)_0.35_PbBr_3_ by dissolving 5 mg of perovskite powder in 0.5 mL of (CD_3_)_2_SO (*d*_6_-DMSO). ^1^H NMR experiments were performed on a 400 MHz Bruker Ascend magnet and Avance III HD NMR. The working frequency for ^1^H NMR was 400 MHz. ^1^H NMR resonances were referenced using tetramethylsilane.

## Results and Discussion

Both end members ternaries of the Cs_*x*_(EtNH_3_)_1–*x*_PbBr_3_ solid solution series do not form a cubic structure and crystallize in either monoclinic crystal system (CsPbBr_3_)^33,49^ or in a 2D perovskite crystal structure ((EtNH_3_)_4_Pb_3_Br_10_).^50^ These structural changes are caused by a mismatch between the sizes of the *A* cation and the lead bromide scaffold, with the Cs^+^ cation being too small (*r* = 1.88 Å)^51^ and EtNH_3_^+^ cation too large to efficiently fill the pores in the perovskite framework. Although the size determination of the ethylammonium cation is more ambiguous, Cheetham et al.^52^ proposed a method that used effective ionic radii, *r*_Aeff_ = *r*_mass_ + *r*_ion_, where *r*_mass_ is the distance from the center of mass of the molecule to the furthest non-hydrogen atom in the molecule, and *r*_ion_ is the Shannon ionic radius of the nitride (N^3–^) anion, which is 1.46 Å. The resulting ionic radius for EtNH_3_^+^ is 2.74 Å,^52^ which is larger than the 2.64 Å limit for 3D perovskite structures, agreeing well with the formation of ((EtNH_3_)_4_Pb_3_Br_10_) phase with reduced dimensionality.^53,54^ Assuming these cation radii, one can employ the Goldschmidt tolerance factor $t=\frac{r_{A}+r_{X}}{\sqrt{2}\left(r_{B}+r_{X}\right)}$ (*r*_*A*_, *r*_*B*_, and *r*_*X*_ are the ionic radii of the *A* site cation, *B* site cation, and *X* site anion, respectively) as a convenient tool for predicting structural stability in perovskite materials.^55^ For stable cubic perovskite structures, the Goldschmidt tolerance factor typically ranges between 0.9 and 1. Due to the limited choices of *A* site cations, alloying offers an effective approach to create such a “perfect sized” *A* cation by tuning the average effective size.^56,57^

We calculated the tolerance factor *t* for Cs_*x*_(EtNH_3_)_1–*x*_PbBr_3_ solid solutions using *r*_*A*_ = *x*·*r*_Cs_ + (1–*x*)·*r*_EtNH3_, where 0 ≤ *x* ≤ 1, *r*_*B*_ = 1.19 Å, and *r*_*X*_ = 1.96 Å to predict the compositions that yield a cubic structure (Figure 1a). The calculations show that the tolerance factor remains within this ideal range when the cesium content in the Cs_*x*_(EtNH_3_)_1–*x*_PbBr_3_ composition remains between 0.29 and 0.8.

**Figure 1:**
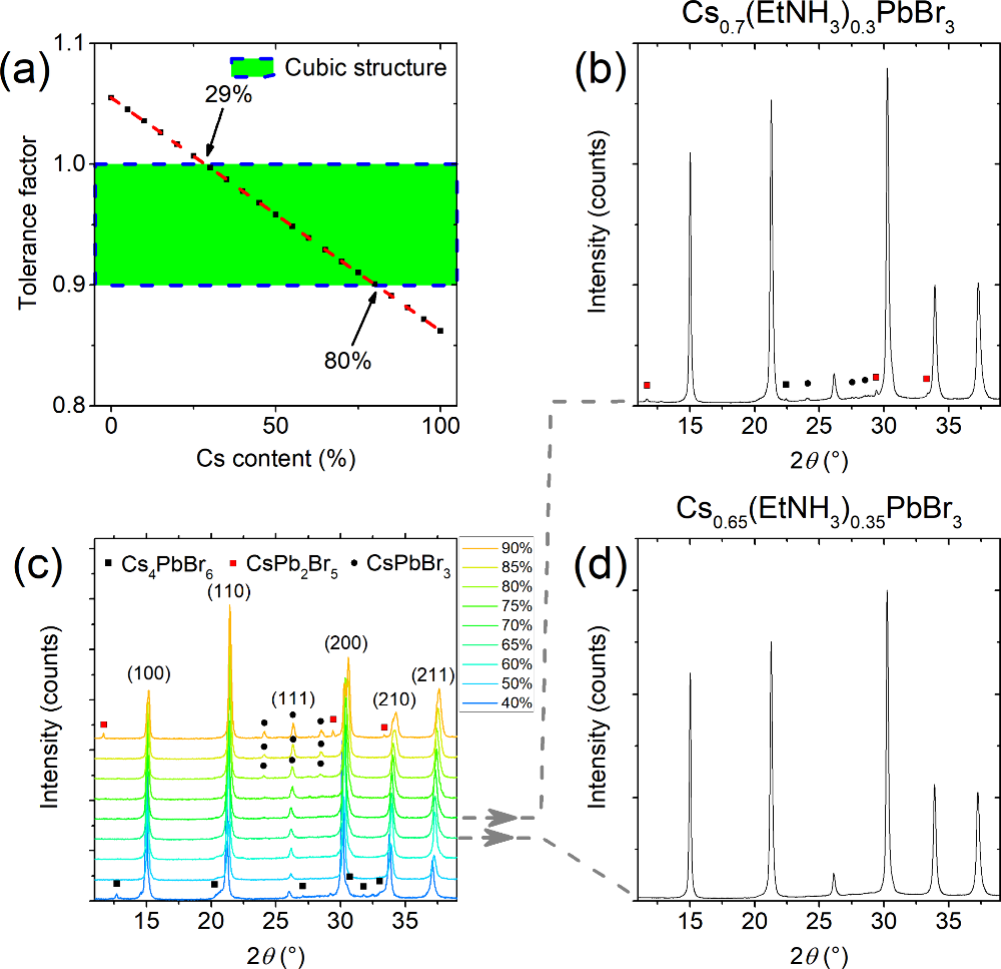
(a) Calculated tolerance factor for the Cs_*x*_(EtNH_3_)_1–*x*_PbBr_3_ solid solutions. (b) Powder X-ray diffraction (PXRD) pattern of the 70% Cs sample. (c) PXRD patterns of Cs_*x*_(EtNH_3_)_1–*x*_PbBr_3_ (*x* = 0.4–0.9) solid solutions. (d) PXRD pattern of the 65% Cs sample. Single crystal data for CsPbBr_3_, CsPb_2_Br_5_, and Cs_4_PbBr_6_ identification were taken from ICSD entries ICSD-143617, ICSD-254290, and ICSD-162158, respectively.^58–60^

To test the stability of Cs_*x*_(EtNH_3_)_1–*x*_PbBr_3_ solid solutions, we performed high-temperature solid-state synthesis of the samples with selected compositions. Synthesis of the solid solutions was conducted by grinding and annealing mixtures of CsBr, EtNH_3_Br, and PbBr_2_ in three steps, with PXRD measurements taken after each step until the formation of a phase pure products was confirmed. To detect the presence of the cubic phase, a peak fitting analysis was performed by comparison with the PXRD pattern of the CH_3_NH_3_PbBr_3_ cubic phase structure,^61^ emphasizing the symmetry and the absence of peak splitting. The cubic phase features no diffraction peaks in a range between 22 and 25°, which can serve as an indication of the target phase formation. There is no indication of competing 2D phases, which usually show strong peaks at lower angles, 3–5° (Figure S1). The samples with higher cesium content, ranging from 40% to 90%, were prepared; however, compositions with cesium content outside the 50–75% range exhibit apparent structural distortions or side products formation (Figure 1c). For instance, a whole profile fitting of the 40% Cs sample indicated that it contains 5.6% of Cs_4_PbBr_6_ side product, whereas the 90% Cs sample contains 47.5% of CsPbBr_3_ and 1.6% of CsPb_2_Br_5_ side phases. For cubic phases, lattice parameters *a* were determined from the PXRD pattern fittings (Figure S2). The results of refinements show an expected gradual decrease of the unit cell size with increasing Cs content (Figure S3), from 5.9424(1) Å for Cs_0.50_(EtNH_3_)_0.50_PbBr_3_ to approximately 5.8951(1) Å for Cs_0.75_(EtNH_3_)_0.25_PbBr_3_, which is in a good agreement with Vegard’s law. The linear change in the unit cell parameter indicates the successful formation of solid solutions without phase separation.

Longer PXRD scans of the compositions with 65% and 70% cesium content were performed to verify the purity of the sample and detect any noncubic phase. The refinements (Figures S4 and S5) confirmed that the 65% cesium solid solution forms a cubic phase, however, the PXRD pattern of the 70% cesium solid solution shows very weak reflections at 12, 22, 24, 28, 30, and 33° that indicate the presence of a noncubic perovskite phase along with CsPb_2_Br_5_ and Cs_4_PbBr_6_ side products (Figure 1b,d). Thus, further characterization was performed for the compositions with 60% and 65% cesium content. The narrow range of the obtained stable cubic phases of Cs_*x*_(EtNH_3_)_1–*x*_PbBr_3_ is likely associated with the uncertainty in the radius of EtNH_3_^+^ cation, although the moisture instability of the EtNH_3_^+^-containing phase can play a role.^56^

We performed the stability test under both inert and air atmospheres (Figure 2). The low cesium content sample Cs_0.50_(EtNH_3_)_0.50_PbBr_3_ demonstrated high stability in an inert atmosphere, showing no change in PXRD patterns upon storage in an Ar-filled glovebox for over a month (Figure 2a). However, sample exposure to air resulted in a rapid, less than 30 min, onset of its decomposition with the formation of CsPb_2_Br_5_. As expected, increasing the Cs content in the samples improves the sample stability toward degradation. For instance, the 60% Cs sample showed the formation of CsPb_2_Br_5_ in under 6 h in the air, whereas the samples with 65% and 70% Cs demonstrated decomposition onset only between 8 and 24 h. The noncubic Cs_0.75_(EtNH_3_)_0.25_PbBr_3_ solid solution showed the onset of its decomposition after 1 h in the air, forming CsPbBr_3_ and CsPb_2_Br_5_, indicating its increased instability compared to the cubic phase (Figure S6). Thus, all compositions showed drastically different stability toward moisture. Air stability tests, based on PXRD measurements for 14 days, indicated that compositions with 65% to 70% cesium content had the best resistance to degradation to CsPb_2_Br_5_, Cs_4_PbBr_6_, and (EtNH_3_)_4_Pb_3_Br_10_. This demonstrates the increased stability of these compositions due to the higher cesium content in these solid solutions.

**Figure 2:**
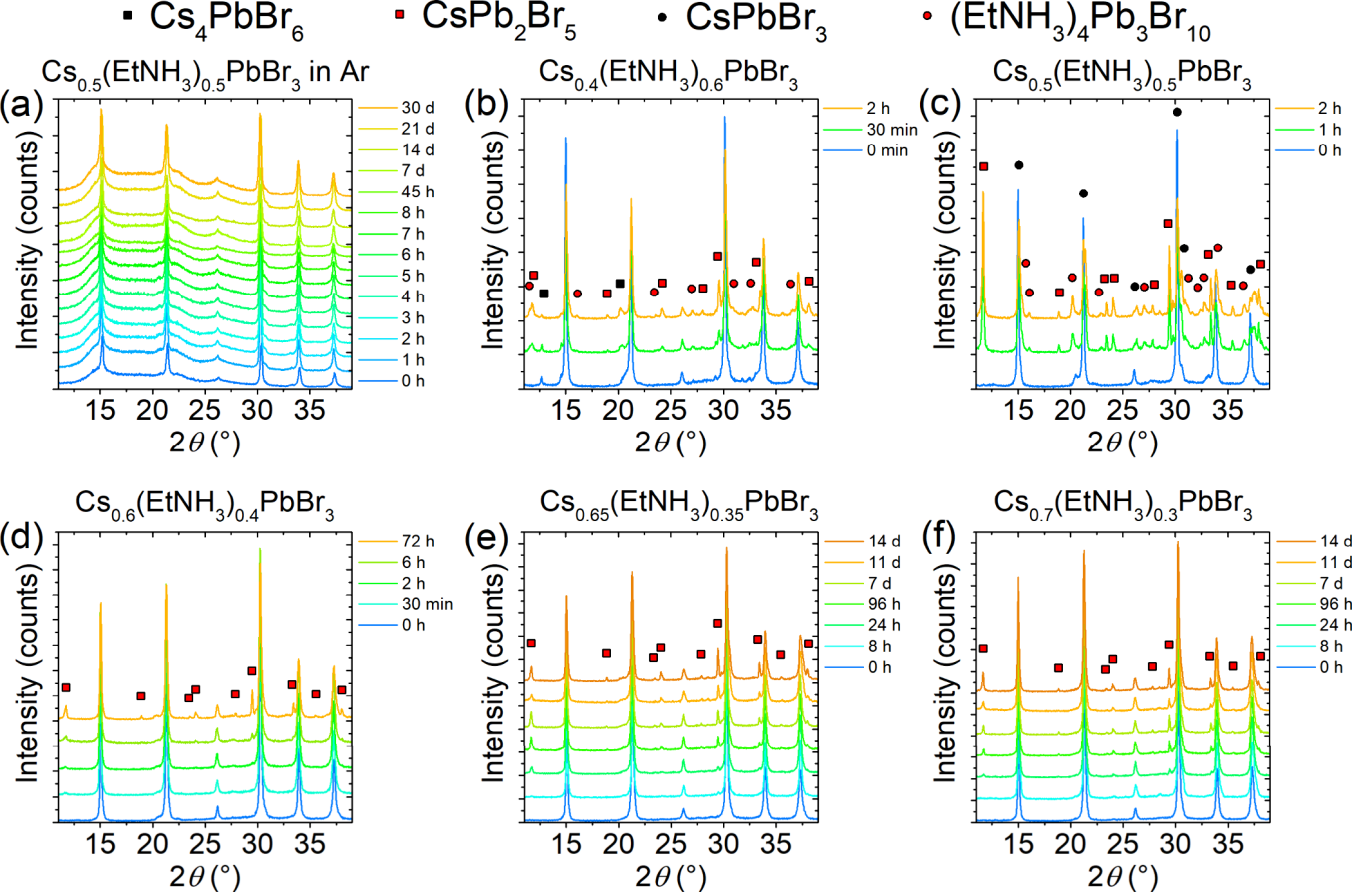
PXRD patterns of Cs_*x*_(EtNH_3_)_1–*x*_PbBr_3_ solid solutions with (a) 50% Cs in an Ar atmosphere; (b) 40% Cs, (c) 50% Cs, (d) 60% Cs, (e) 65% Cs, and (f) 70% Cs exposed to air over different periods of time.

Scanning electron microscopy (SEM) and energy-dispersive X-ray spectroscopy (EDS) were used to investigate the morphology and elemental composition of the synthesized solid solutions, as shown in Figure 3. EDS mapping confirmed the uniform distribution of cesium, lead, and bromine in the samples, supporting the homogeneity of the solid solution phase. The elemental analysis shows the following atomic percentages for the 60% and 65% cesium samples: 13.3% Cs, 22.2% Pb, and 64.5% Br for the 60% sample; and 14.1% Cs, 22.1% Pb, and 63.8% Br for the 65% sample. These values correspond to the compositions of Cs_0.6_(EtNH_3_)_0.4_PbBr_2.91_ and Cs_0.64_(EtNH_3_)_0.36_PbBr_2.89_, respectively, confirming the expected stoichiometry and validating the accuracy of the synthetic methods. It is noteworthy that the slight Br substoichiometry may indicate halide defects in the cubic structure, similar to previously reported “hollow” perovskite.^62^ However, this cannot be confirmed based solely on EDS data due to its semiquantitative nature.

**Figure 3:**
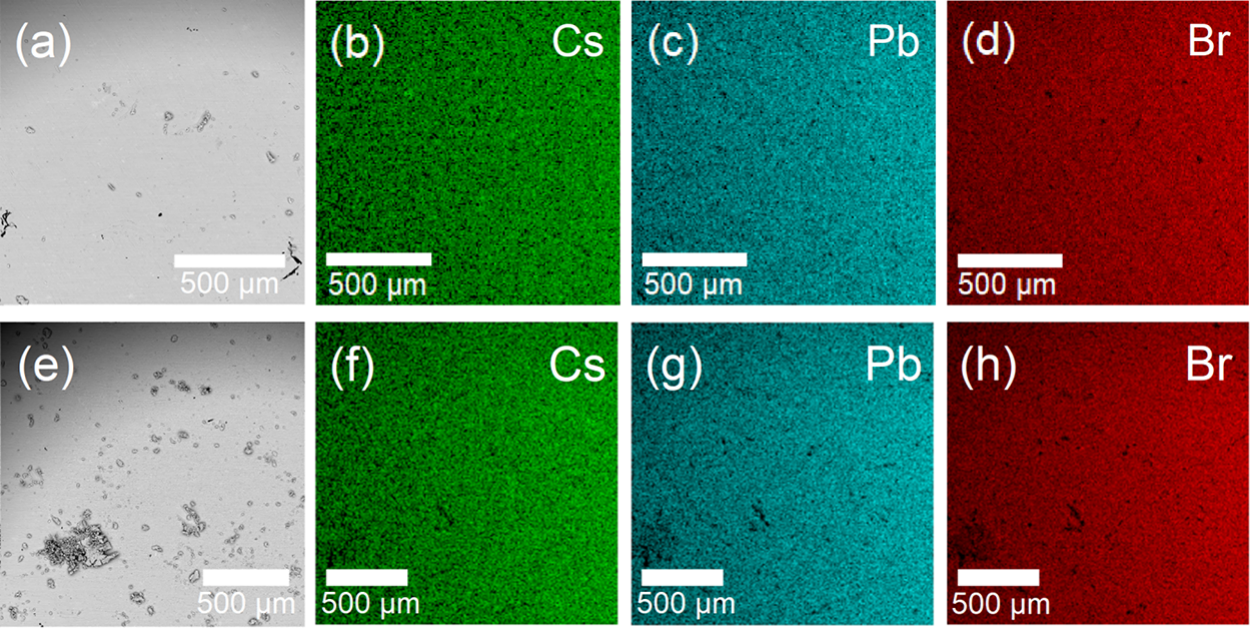
SEM images and EDS elemental mappings of (a–d) Cs_0.6_(EtNH_3_)_0.4_PbBr_3_ and (e–h) Cs_0.65_(EtNH_3_)_0.35_PbBr_3_.

To confirm the incorporation of ethylammonium into the products, we collected ^1^H NMR and vibrational spectra of the 60% and 65% Cs samples. The NMR spectra of the samples dissolved in *d*_6_-DMSO showed a singlet at 7.62–7.66 ppm, a quartet at 2.9 ppm, and a triplet at 1.21 ppm (Figure S7) corresponding to chemical shifts from −NH_3_^+^, −CH_2_– and −CH_3_ protons, respectively. The IR and Raman spectra (Figure 4 and Figure S8) exhibit stretching and bending modes characteristic of ethylammonium cations (Tables S1 and S2). The IR spectra recorded in the range of 400–4000 cm^–1^ (Figure 4), contain bands indicating organic cation vibrations: C–H, N–H, C–C and C–N stretching bonds, H–C–H, H–N–H and C–C–N bending bonds, NH_3_ rocking and CH_2_ twisting bonds (Table S1). The Raman spectra collected in the range of 200–2000 cm^–1^ (Figure S8) show peaks from C–N and C–C stretching bonds, CH_3_, NH_3_ and C–C–N bending bonds (Table S2).^63^

**Figure 4:**
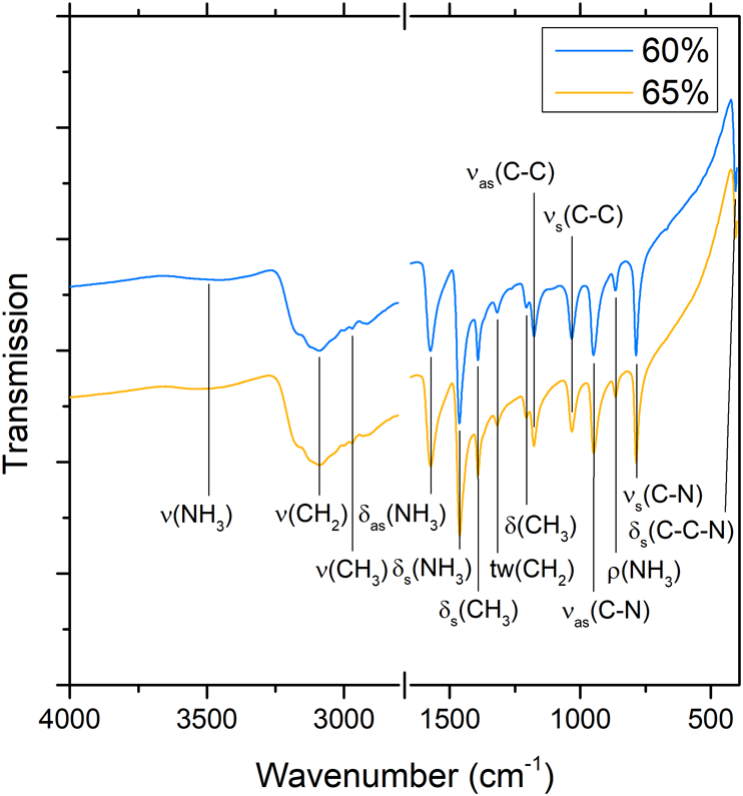
IR spectra of Cs_0.6_(EtNH_3_)_0.4_PbBr_3_ and Cs_0.65_(EtNH_3_)_0.35_PbBr_3_ at room temperature in the 400–4000 cm^–1^ range.

UV–vis absorption measurements were used to determine the optical bandgaps of the samples. The obtained reflectance spectra were transformed to the corresponding Tauc plots by applying the Kubelka–Munk function.^48^ The plots show bandgaps of 2.28 and 2.27 eV for the 60% and 65% of cesium samples, respectively (Figure 5). These values are similar to those for pure CsPbBr_3_, which range from 2.25 to 2.29 eV,^32,64,65^ indicating that the incorporation of the ethylammonium cation has little effect on the electronic energy states of the perovskite, similar to other organic cations.^66,67^

**Figure 5:**
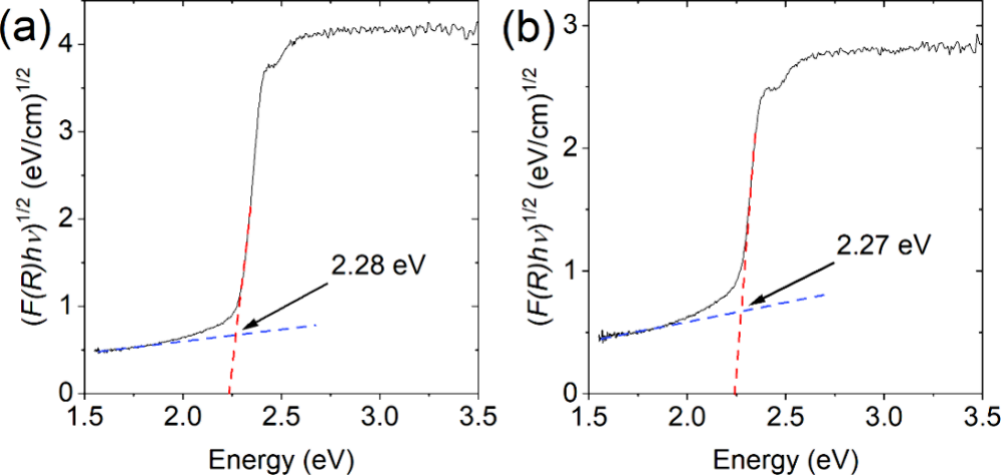
Tauc plots of (a) Cs_0.6_(EtNH_3_)_0.4_PbBr_3_ and (b) Cs_0.65_(EtNH_3_)_0.35_PbBr_3_ obtained using the Kubelka–Munk function to determine the bandgap.

Electrical properties of the powder materials were measured by collecting I–V curves from 60% and 65% Cs samples. Devices fabricated using Au/Cs_0.6_(EtNH_3_)_0.4_PbBr_3_/Au and Au/Cs_0.65_(EtNH_3_)_0.35_PbBr_3_/Au configurations showed a linear I–V dependence with ohmic behavior at voltages from −300 to 300 V (Figure 6). The calculated resistivity values are 40.1 MΩ·cm and 27.4 MΩ·cm, respectively, which meet the requirement of high resistivity for radiation detection.^68^

**Figure 6:**
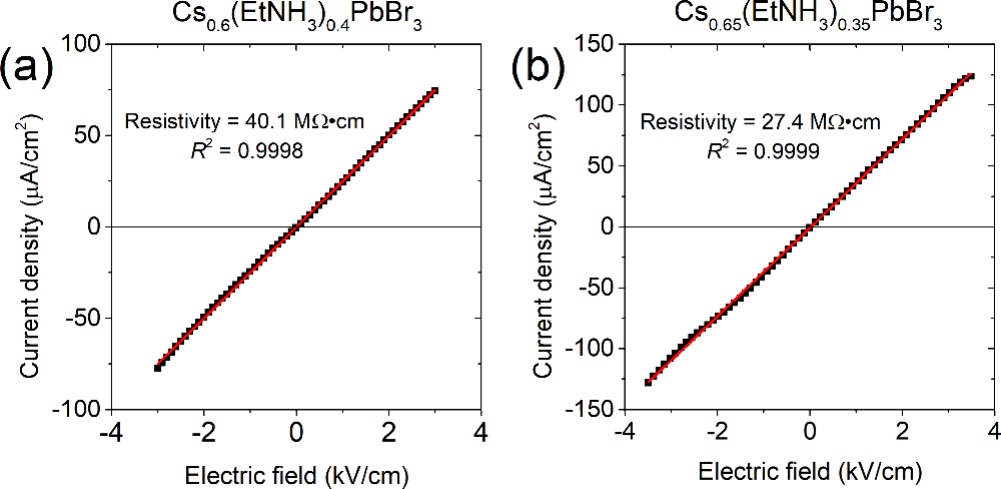
I–V curves of (a) Au/Cs_0.6_(EtNH_3_)_0.4_PbBr_3_/Au and (b) Au/Cs_0.65_(EtNH_3_)_0.35_PbBr_3_/Au devices.

Photoluminescence (PL) measurements on the Cs_0.65_(EtNH_3_)_0.35_PbBr_3_ sample showed no apparent excitation peaks in the 240–450 nm range and three broad emission peaks in the 400–750 nm range (Figure S9). The peak at 530 nm is attributable to the CsPbBr_3_ band edge emission, but the nature of the remaining two peaks is not as clear, although they are likely due to defect states. Additionally, we tested the X-ray sensitivity of the devices, in which no photocurrent relative to the dark current was observed. These responses demonstrated the low sensitivity of the devices, which is attributed to the high concentration of defects that are primarily associated with grain boundaries.^69^ Given that we used the solid-state synthesis method to obtain solid solutions in the form of powders and pressed pellets, one would expect a high density of grains and pores, which worsened the results of PL and X-ray sensitivity measurements. To improve PL response and X-ray sensitivity, alternative synthesis methods, such as single crystal growth or thin film deposition, should be explored to produce higher quality Cs_*x*_(EtNH_3_)_1–*x*_PbBr_3_ solid solutions. However, our attempts to synthesize Cs_*x*_(EtNH_3_)_1–*x*_PbBr_3_ single crystals using several solution-based methods, such as cooling crystallization (CC), inverse temperature crystallization (ITC), and antisolvent vapor-assisted crystallization (AVC), were not successful (Table S3). The CC method yielded two phases of CsPbBr_3_ and (EtNH_3_)_4_Pb_3_Br_10_, whereas the ITC and AVC methods resulted in only CsPbBr_3_ precipitated without the formation of phases containing ethylammonium. The synthesis of single crystals can likely be achieved through the use of high boiling point solvents that will promote sample crystallization at elevated temperatures.

## Conclusions

In this report, we prepared a series of Cs_*x*_(EtNH_3_)_1–*x*_PbBr_3_ solid solutions and tested their stability. The results demonstrate the successful incorporation of EtNH_3_^+^ into the crystal lattice, which stabilizes the cubic phase of the perovskite in the compositional range of Cs_0.5_(EtNH_3_)_0.5_PbBr_3_ to Cs_0.65_(EtNH_3_)_0.35_PbBr_3_. Due to the large size of the ethylammonium cation, more cesium can be accommodated in the solid solution to obtain the desired cubic perovskite structure. In addition, solid solutions with higher Cs content show increased resistance to phase degradation in air. UV–vis absorption studies indicate a bandgap of 2.27–2.28 eV, which is similar to pure CsPbBr_3_ phase, indicating a negligible impact of the ethylammonium cation on the bandgap width. Devices with different cesium contents showed high resistivity of Cs_*x*_(EtNH_3_)_1–*x*_PbBr_3_ perovskites on the order of 10^7^ Ω·cm. We anticipate that further advancements in device performance can be achieved by modifying the synthetic methods to obtain high-quality single crystals, which will exhibit improved optoelectronic properties and sufficient sensitivity for radiation detection.

## Supplementary Material



## Notes

Additional details regarding assignment of the IR and Raman bands, reaction conditions used for solution growth methods, lattice parameter dependence on the cesium content, Le Bail fitting plots, PXRD patterns of 75% Cs sample exposed to air, ^1^H NMR spectra, Raman spectra, and PL emission and excitation spectra (PDF)
