# Supplementary material for: Cubic Cesium Lead Bromide Stabilized by Ethylammonium Incorporation
Source: Inorg Chem. 2025 Aug 5;64(32):16433–40. doi: 10.1021/acs.inorgchem.5c02183 (PMC12365875; doi:10.1021/acs.inorgchem.5c02183)
Supplement: Supplementary file 1 [file ic5c02183_si_001.pdf]

Supporting information for

Cubic Cesium Lead Bromide Stabilized by Ethylammonium Incorporation

Maksim Tabatadze,[a] Aleksandra D. Valueva,[a] Hope A. Long,[a] Sergei A. Novikov,[a] Yiping Zhao,[b] and Vladislav V. Klepov*[a]

[a]Department of Chemistry, University of Georgia, Athens, Georgia, 30602, United States

[b]Department of Physics and Astronomy, University of Georgia, Athens, Georgia, 30602, United States

*Corresponding author. E-mail: klepov@uga.edu

Table S1. Assignment of the IR bands for $Cs_{0.6}(EtNH_3)_{0.4}PbBr_3$ and $Cs_{0.65}(EtNH_3)_{0.35}PbBr_3$[1]

Wavenumber, cm^{-1}		Assignment
$Cs_{0.6}(EtNH_3)_{0.4}PbBr_3$	$Cs_{0.65}(EtNH_3)_{0.35}PbBr_3$	
405 m	405 m	δ_s(C-C-N)
786 s	786 s	ν_s(C-N)
865 m	865 m	$\rho(NH_3)$
949 s	949 s	ν_{as}(C-N)
1033 s	1032 s	ν_s(C-C)
1177 s	1177 s	ν_{as}(C-C)
1206 m	1206 m	$\delta(CH_3)$
1318 m	1318 m	tw(CH_2)
1392 s	1391 s	$\delta_s(CH_3)$
1463 s	1461 s	$\delta_s(NH_3)$
1573 s	1573 s	$\delta_{as}(NH_3)$
2970 w	2971 w	$\nu(CH_3)$
3091 m	3090 m	$\nu(CH_2)$
3454 w	3547 w	$\nu(NH_3)$

* s: strong, m: medium, w: weak

Table S2. Assignment of the Raman bands for $Cs_{0.6}(EtNH_3)_{0.4}PbBr_3$ and $Cs_{0.65}(EtNH_3)_{0.35}PbBr_3$

Wavenumber, cm^{-1}		Assignment
$Cs_{0.6}(EtNH_3)_{0.4}PbBr_3$	$Cs_{0.65}(EtNH_3)_{0.35}PbBr_3$	
229 s<br>311 s<br>403 w	229 s<br>312 s<br>405 m	δ(C-C-N)
869 m	870 m	ν_{as}(C-N)
956 w	950 w	ν(C-C)
1188 w	1185 w	ν_{as}(C-C)
1329 w	1327 w	$\delta_s(CH_3)$
1458 w<br>1476 w	1460 w<br>1474 w	$\delta_{as}(CH_3)$
1582 w	1577 w	$\delta_{as}(NH_3)$

* s: strong, m: medium, w: weak

Table S3. Reaction conditions used for crystal solution growth methods.

CsBr	$C_2H_5NH_3Br$	$PbBr_2$	Solvent	Method
1 M	1 M	4 M	DMSO	ITC
1 M	2 M	4 M	DMSO	ITC
1 M	4 M	4 M	DMSO	ITC
0.34 M	2.69 M	2.69 M	DMSO	ITC
0.11 M	0.42 M	0.21 M	HBr	CC
0.11 M	0.84 M	0.21 M	HBr	CC
0.11 M	0.42 M	0.11 M	HBr	CC
0.11 M	0.84 M	0.11 M	HBr	CC
1 M	3 M	4 M	DMSO : DMF = 1:1	AVC
1 M	3 M	4 M	DMSO : GBL = 1:3	AVC
0.45 M	1.35 M	1.8 M	DMSO	Hydrothermal at 200 ˚C
0.1 M	0.1 M	0.2 M	GBL	Hydrothermal at 200 ˚C
0.05 M	0.2 M	0.2 M	GBL	Hydrothermal at 220 ˚C
0.05 M	0.2 M	0.1 M	GBL	Hydrothermal at 220 ˚C

*ITC – inverse temperature crystallization; CC – cooling crystallization; AVC – anti-solvent vapor-assisted crystallization methods

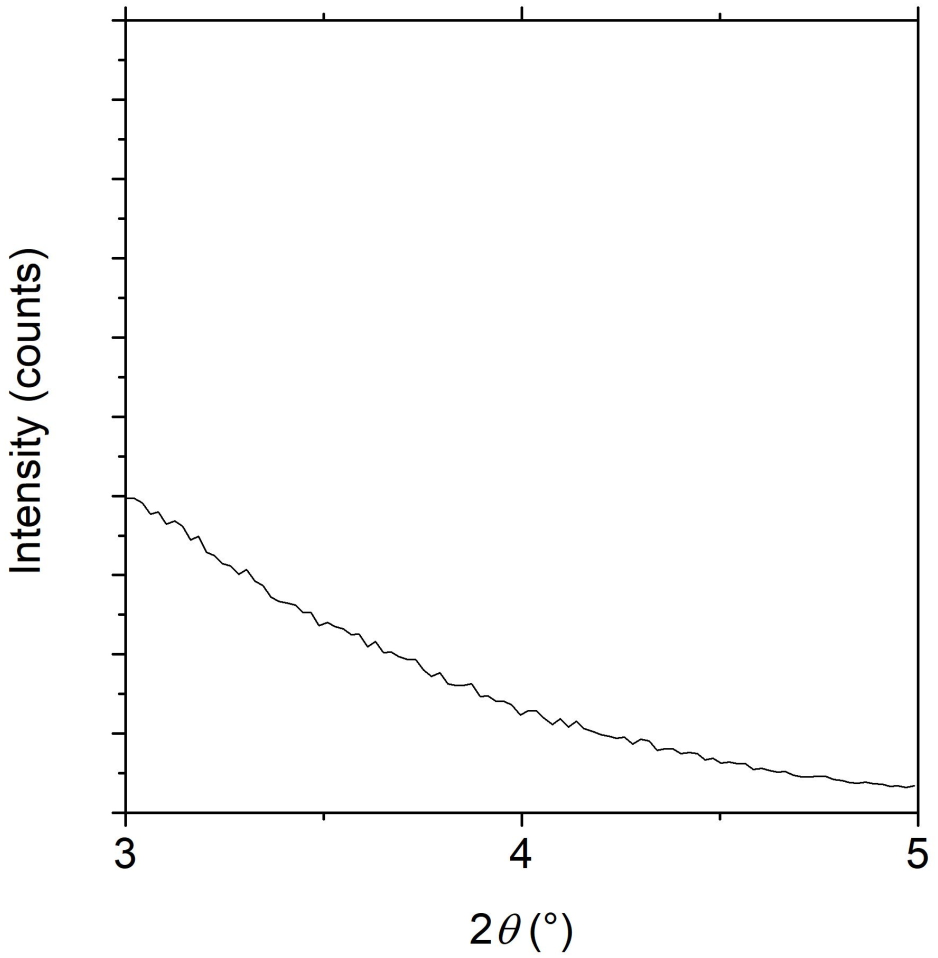

Figure S1. PXRD pattern of $Cs_{0.65}(EtNH_3)_{0.35}PbBr_3$ at 3–5° range.

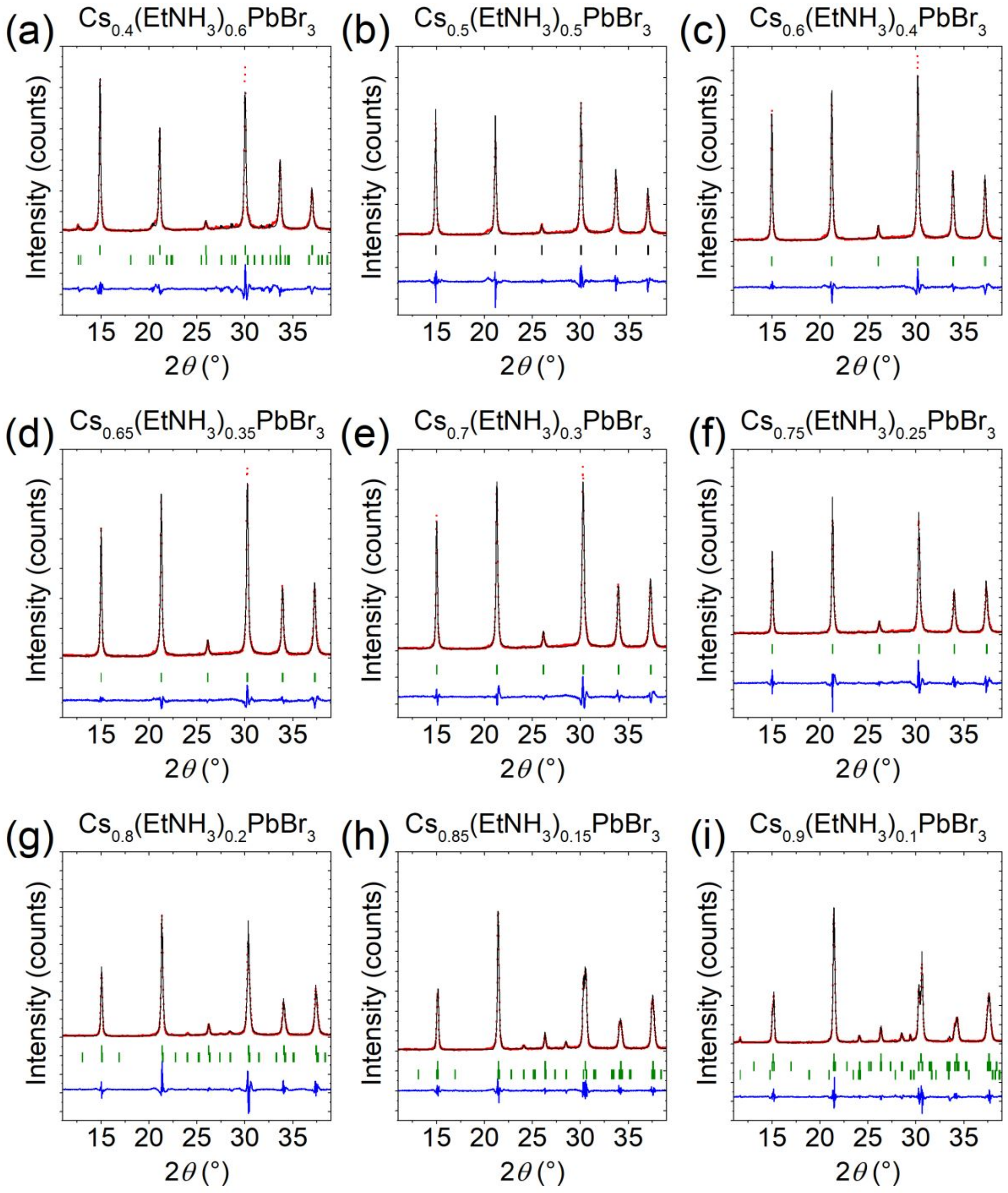

Figure S2. Le Bail fit on PXRD patterns of (a) 40% Cs, (b) 50% Cs, (c) 60% Cs, (d) 65% Cs, (e) 70% Cs, (f) 75% Cs, (g) 80% Cs, (h) 85% Cs, and (i) 90% Cs samples.

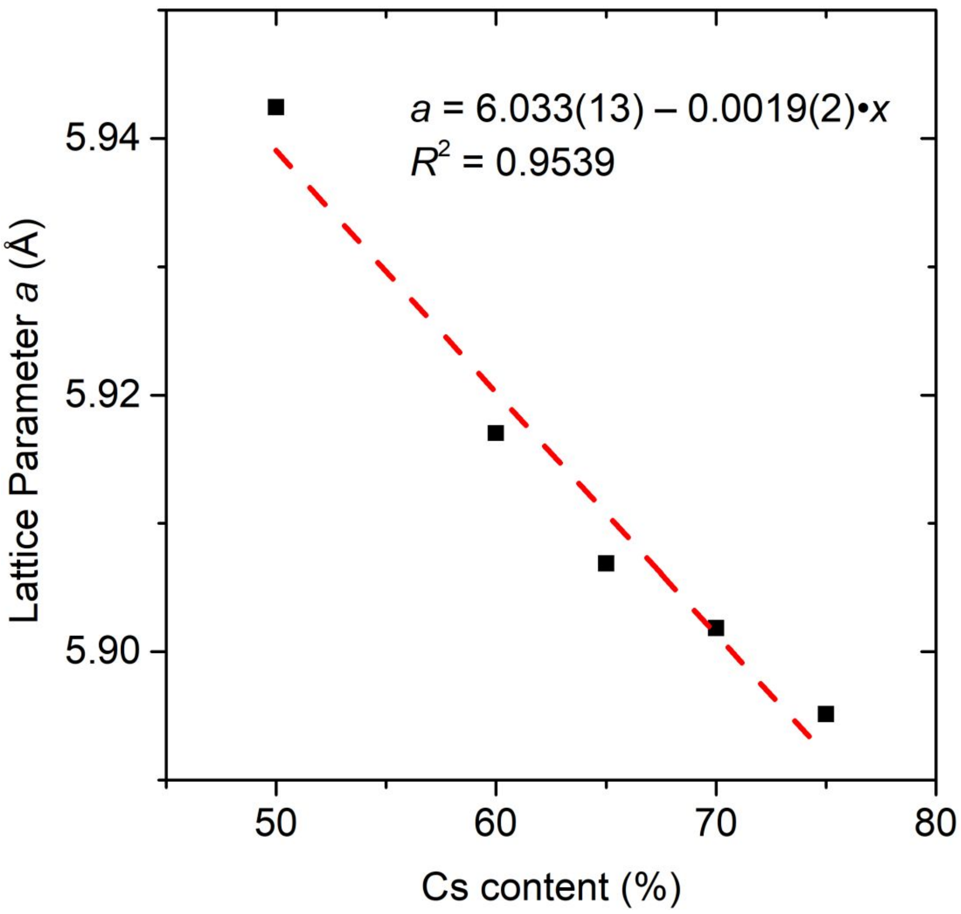

Figure S3. The dependence of the lattice parameter on the cesium content in $Cs_x(EtNH_3)_{1-x}PbBr_3$ follows the empirical linear trend of Vegard's law.

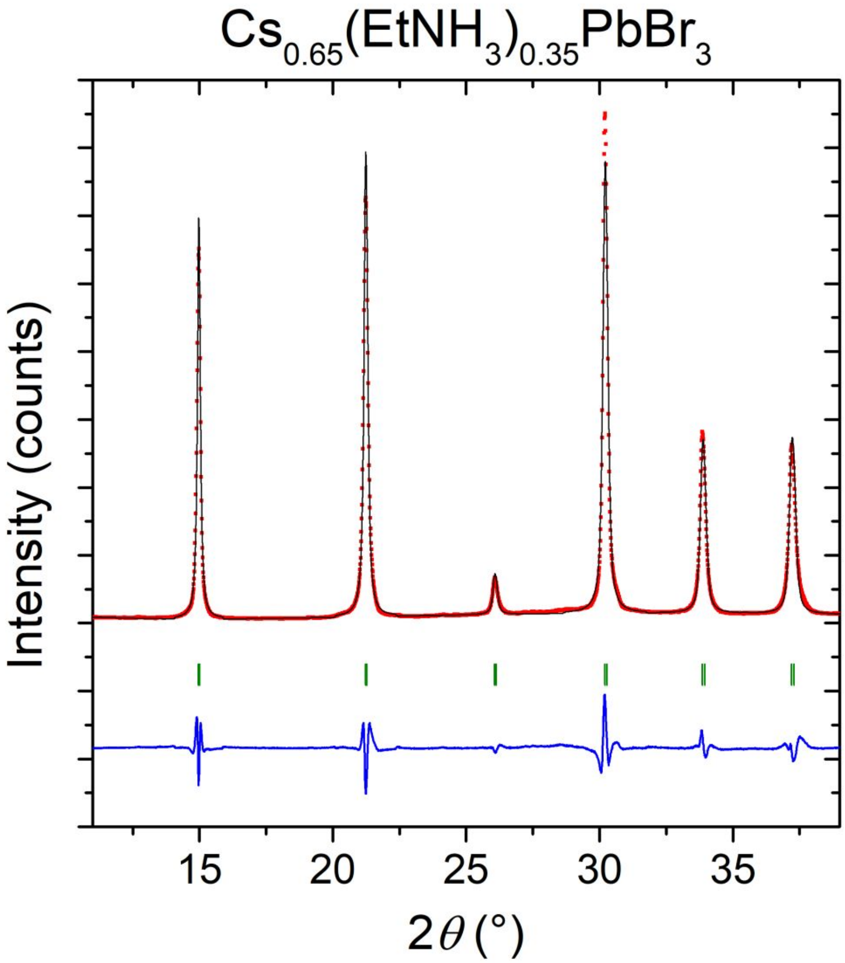

Figure S4. Le Bail fit on long scanned PXRD of $Cs_{0.65}(EtNH_3)_{0.35}PbBr_3$. ($D$ = 7.9783 nm)

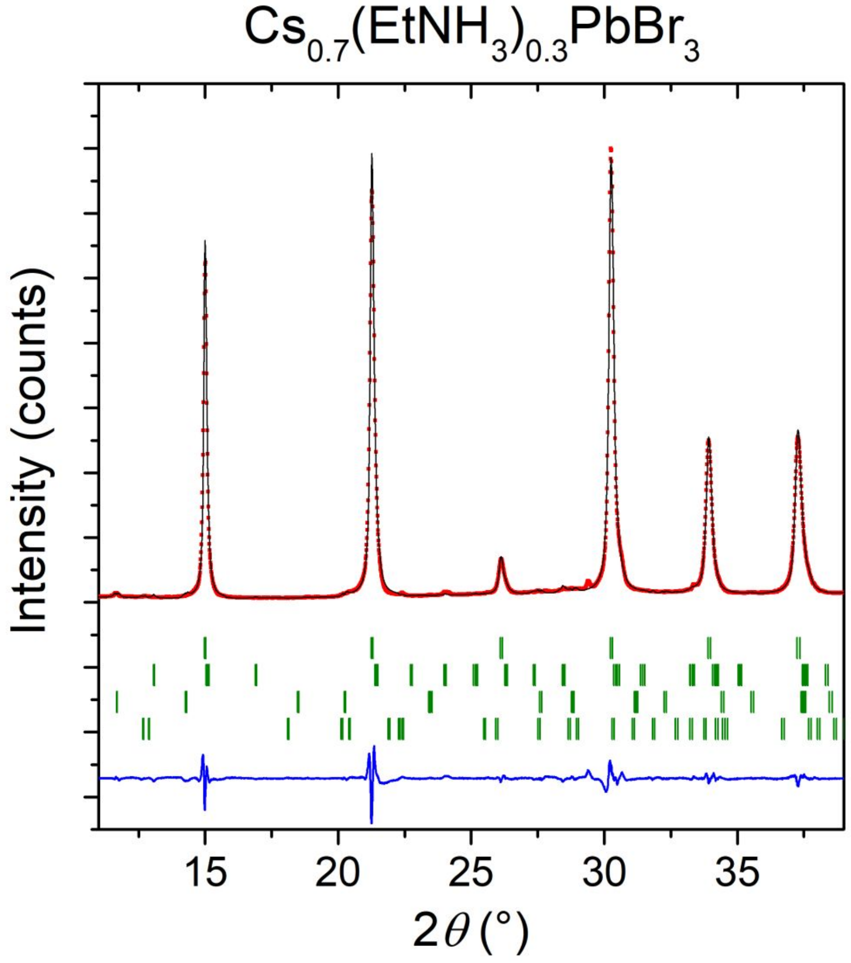

Figure S5. Le Bail fit on long scanned PXRD of $Cs_{0.7}(EtNH_3)_{0.3}PbBr_3$. ($D$ = 9.5396 nm)

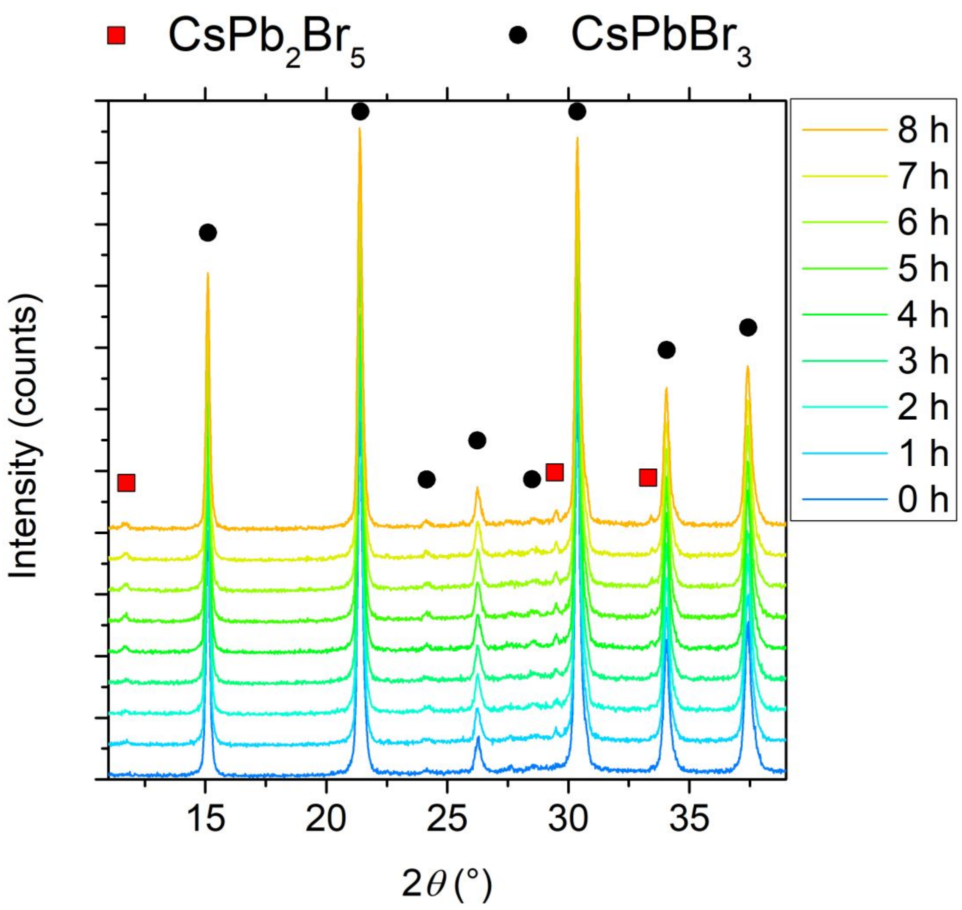

Figure S6. PXRD patterns of $Cs_{0.75}(EtNH_3)_{0.25}PbBr_3$ solid solution exposed to air over 8 hours.

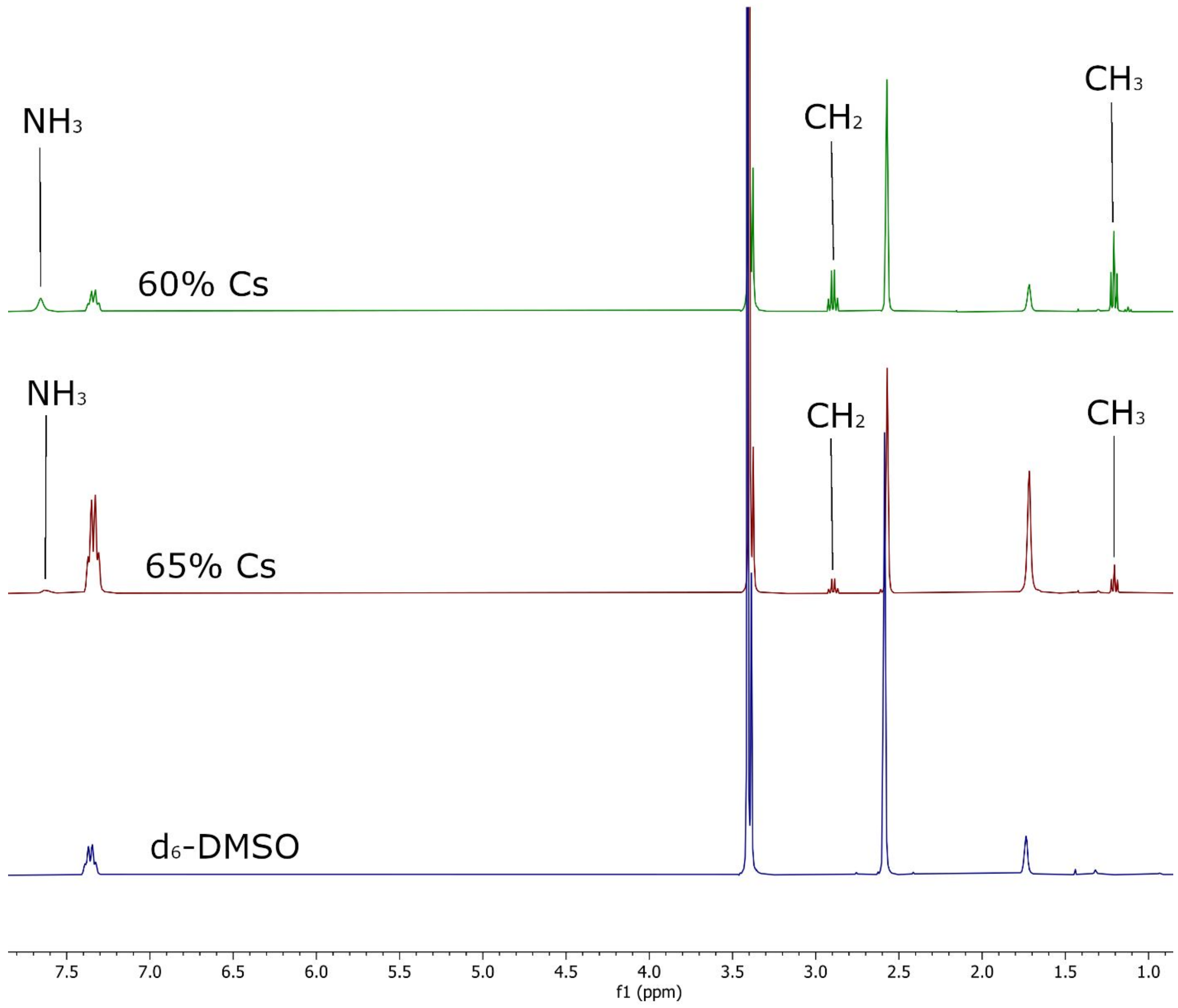

Figure S7. ^{1}H-NMR spectra of $Cs_{0.6}(EtNH_3)_{0.4}PbBr_3$, $Cs_{0.65}(EtNH_3)_{0.35}PbBr_3$, and a pure solvent d_6-DMSO.

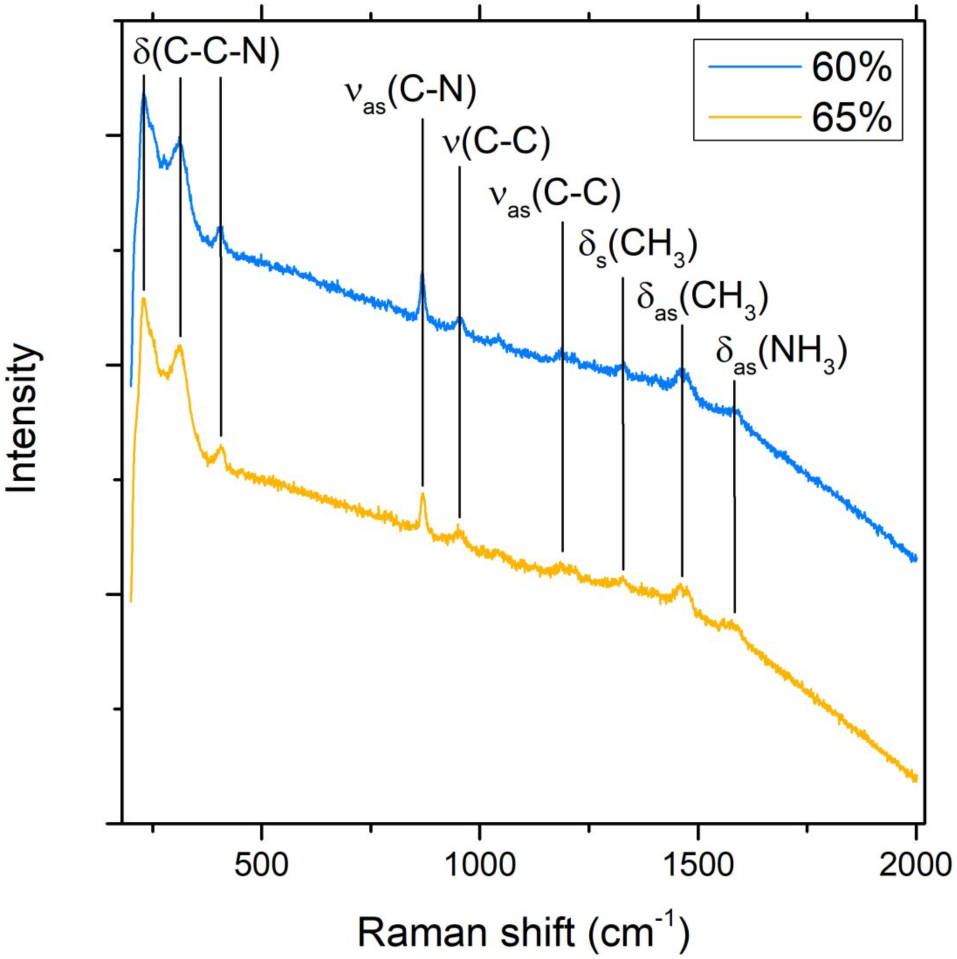

Figure S8. Raman spectra of $Cs_{0.6}(EtNH_3)_{0.4}PbBr_{2.91}$ and $Cs_{0.64}(EtNH_3)_{0.36}PbBr_{2.89}$ at room temperature in the 200–2000 cm^{-1} range.

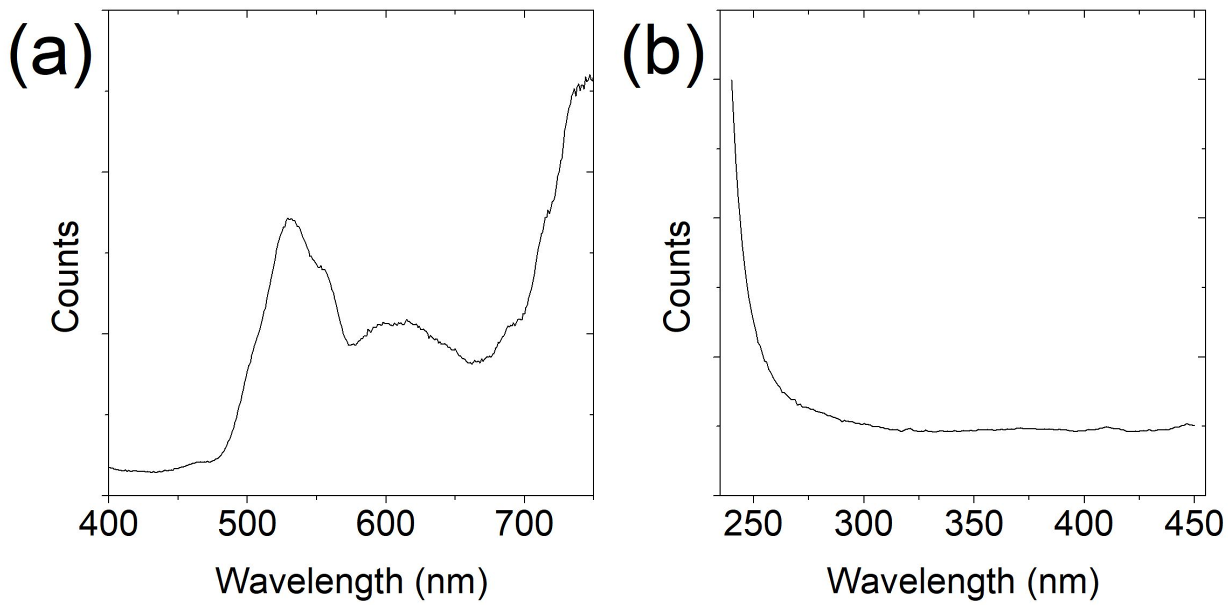

Figure S9. (a) PL emission spectra at 300 nm excitation wavelength, and (b) PL excitation spectra at 530 nm emission wavelength of $Cs_{0.65}(EtNH_3)_{0.35}PbBr_3$.

References

1. Nakamoto, K. *Infrared and Raman spectra of inorganic and coordination compounds, part B: applications in coordination, organometallic, and bioinorganic chemistry*; John Wiley & Sons; 2009. DOI: 10.1002/9780470405840
